# Beyond Fiber: Toward Terahertz Bandwidth in Free-Space Optical Communication

**DOI:** 10.3390/s25072109

**Published:** 2025-03-27

**Authors:** Rahat Ullah, Sibghat Ullah, Jianxin Ren, Hathal Salamah Alwageed, Yaya Mao, Zhipeng Qi, Feng Wang, Suhail Ayoub Khan, Umar Farooq

**Affiliations:** 1 Institute of Optics and Electronics, Nanjing University of Information Science & Technology, Nanjing 210044, China; 003458@nuist.edu.cn (J.R.); maoyy@nuist.edu.cn (Y.M.); 003268@nuist.edu.cn (Z.Q.); 003101@nuist.edu.cn (F.W.); 2 Jiangsu Key Laboratory for Optoelectronic Detection of Atmosphere and Ocean, Nanjing University of Information Science & Technology, Nanjing 210044, China; 3 Jiangsu International Joint Laboratory on Meteorological Photonics and Optoelectronic Detection, Nanjing University of Information Science & Technology, Nanjing 210044, China; 4 School of Electronic Science and Engineering, Southeast University, Nanjing 210018, China; 5 College of Computer and Information Sciences, Jouf University, Sakaka 72388, Saudi Arabia; hswageed@ju.edu.sa; 6 School of Chemistry and Chemical Engineering, Yangzhou University, Yangzhou 225009, China; 008624@yzu.edu.cn; 7 Institute of Advanced Materials and Flexible Electronics (IAMFE), School of Chemistry and Materials Science, Nanjing University of Information Science and Technology, Nanjing 210044, China; 8 Department of Chemistry, School of Basic Sciences, Galgotais University, Greater Noida 201309, India; umar.farooq@galgotiasuniversity.edu.in

**Keywords:** THz-bandwidth, THz communication systems, FSO communication systems, integrated THz-FSO systems, software-defined networking

## Abstract

The rapid advancement of terahertz (THz) communication systems has positioned this technology as a key enabler for next-generation telecommunication networks, including 6G, secure communications, and hybrid wireless-optical systems. This review comprehensively analyzes THz communication, emphasizing its integration with free-space optical (FSO) systems to overcome conventional bandwidth limitations. While THz-FSO technology promises ultra-high data rates, it is significantly affected by atmospheric absorption, particularly absorption beyond 500 GHz, where the attenuation exceeds 100 dB/km, which severely limits its transmission range. However, the presence of a lower-loss transmission window at 680 GHz provides an opportunity for optimized THz-FSO communication. This paper explores recent developments in high-power THz sources, such as quantum cascade lasers, photonic mixers, and free-electron lasers, which facilitate the attainment of ultra-high data rates. Additionally, adaptive optics, machine learning-based beam alignment, and low-loss materials are examined as potential solutions to mitigating signal degradation due to atmospheric absorption. The integration of THz-FSO systems with optical and radio frequency (RF) technologies is assessed within the framework of software-defined networking (SDN) and multi-band adaptive communication, enhancing their reliability and range. Furthermore, this review discusses emerging applications such as self-driving systems in 6G networks, ultra-low latency communication, holographic telepresence, and inter-satellite links. Future research directions include the use of artificial intelligence for network optimization, creating energy-efficient system designs, and quantum encryption to obtain secure THz communications. Despite the severe constraints imposed by atmospheric attenuation, the technology’s power efficiency, and the materials that are used, THz-FSO technology is promising for the field of ultra-fast and secure next-generation networks. Addressing these limitations through hybrid optical-THz architectures, AI-driven adaptation, and advanced waveguides will be critical for the full realization of THz-FSO communication in modern telecommunication infrastructures.

## 1. Introduction

Facing the predicted 396 exabytes of global data traffic per month by 2025, there is a manifest need for communication systems that support high-bandwidth and ultra-latency communication. The health of Ericsson is well documented by its latest report, especially as it relates to growth in the following areas of technology: 5G, autonomous vehicles, the IoT, and high-definition video streaming [1]. Currently, communication systems, such as fiber-optic systems, have been effective. Still, these systems have some constraints because they require stationary systems and may not be helpful under certain circumstances [2]. Free-space optical (FSO) communication, also referred to as wireless optical communications, has increasingly attracted interest as a method for high-speed wireless communication that does not require wires or fiber optic systems [3,4]. FSO systems work in the visible and near-IR ranges and can thus transmit at speeds from Gbps to Tbps when conditions are favorable.

Nevertheless, due to the direct dependence of this method on the atmospheric conditions, the system can be easily disturbed by the presence of fog, rain, or other turbulences, which negatively impact the system’s performance [5,6]. Traditional FSO systems have certain drawbacks, which have led to the development of a suggested terahertz (THz) band range of 0.1–10 THz. The THz band has over 10 times the bandwidth of 100 GHz millimeter-wave (mmWave) communication and provides data rates that exceed 1 Tbps, providing connectivity between microwave and optical frequencies [7,8]. Other advantages of shorter wavelengths in the THs spectrum include their compactness and power efficiency, which are typical requirements for portable or space-demanding technologies in various applications [3,9]. However, multiple hurdles must be overcome before THz communication technology can be fully adopted. Signal losses over 15 dB/km occur due to atmospheric attenuation, which is mainly caused by water vapor absorption bands [4]. Another essential feature is the line-of-sight alignment, because even a tiny deviation concerning this parameter can lead to considerable disruptions in data transmission. The low output power of THz sources and the absence of high-sensitivity and inexpensive THz detectors make it difficult to implement the THz-FSO system [10,11]. Recent developments in photonic technologies like QCLs and metamaterial-based gadgets have enhanced the characteristics of THz systems [12,13]. Hence, adaptive optics and machine learning algorithms for achieving alignment are also being implemented to counteract the influence of turbulence in maintaining a reliable communication connection. Such advancements point to the chances of incorporating the THz frequency within FSO systems to improve a new generation of dense networks [11].

**Figure 1:**
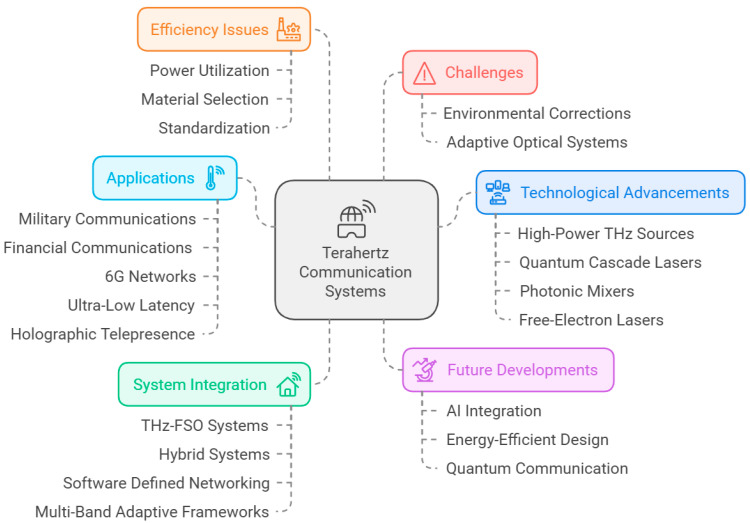
Toward terahertz bandwidth in free-space optical communication.

Figure 1 shows different aspects of toward terahertz bandwidth in free-space optical communication. The uses of THz-FSO communication are as follows: inter-satellite communication, high-speed backhaul for 5G communication networks, and military communication [6,14]. THz-FSO links benefit from a fairly narrow beam width, making those used for critical applications much safer since interception is unlikely. Furthermore, THz-FSO systems, due to their low latency and high bandwidth, are applicable to upcoming technologies such as 6G wireless networks and car-to-car data transfer [15,16]. This review article presents THz-based FSO communication alongside theoretical fundamentals, relevant enabling technologies, and the issues that are confronted in this promising field. This paper aims to establish how THz-FSO communication can be the solution to fulfilling the requirements of future networks by combining and synthesizing the most relevant discoveries from the literature [6,14,15,16].

### 1.1. Objectives and Scope

The purpose of the current review paper is to organize the literature on terahertz-bandwidth free-space optical communication and to investigate the possibilities regarding its role in the evolution of high-speed communication networks. The key objectives are:

- To foresee the fundamental and physical aspects of terahertz frequencies;
- To assess the development of enabling technologies that facilitate the development of THz systems, such as THz sources, detectors, and adaptive systems;
- In this paper, some of the issues regarding this technology, such as future research directions, its massive energy consumption, and its susceptibility to abnormal weather conditions, will also be discussed, with a focus on the limitations and challenges, such as atmospheric attenuation and hardware limitations in implementing THz-FSO systems;
- To analyze THz-FSO communication and other systems, comparisons are made here to microwave and optical systems;
- To explore possible use cases that include inter-satellite links, the sixth generation network, and safe communications.

This review aims to cover the interrelated fields of material chemistry, signal processing, and mixed communication systems. By analyzing more than 100 recently published articles, this paper attempts to present the current state of and findings related to the technological readiness of terahertz-based free-space optical communication and its future outlook.

### 1.2. Comparative Analysis of Communication Systems

The comparative analysis, conducted herein, of the available communication technologies underscores the strengths and weaknesses of microwave-, optical-, and terahertz-based FSO systems. The table below provides a detailed comparison.

Table 1 highlights the prospects of terahertz-bandwidth FSO systems in enhancing ultra-fast communication within the dimensions of bandwidth and security.

This review provides a comprehensive analysis of THz-FSO communication systems, addressing key challenges and advancements that have not been extensively covered in previous surveys. Unlike the existing reviews, which primarily focus on either THz communication or FSO systems separately, our study emphasizes the hybrid integration of THz and FSO technologies to enhance high-speed wireless communication. One of the key contributions of our work is the detailed discussion of the security aspects of these technologies, an area that has received limited attention in THz-FSO research. We examine potential vulnerabilities, interception risks, and the robustness of THz-FSO links against eavesdropping and jamming attacks. Additionally, we provide an in-depth analysis of integration challenges, including hardware constraints, alignment issues, and optimization techniques for hybrid THz-FSO networks. Furthermore, this study introduces new insights into atmospheric absorption models, dispersion effects, and THz device limitations. While previous surveys have briefly mentioned attenuation in regard to THz frequencies, our review quantifies the impact of atmospheric absorption above 500 GHz. It identifies the 680 GHz transmission window as an optimal band for THz-FSO communication. We also provide a detailed discussion on atmospheric dispersion, explaining how humidity, temperature fluctuations, and pressure variations affect THz signal propagation, which leads to pulse broadening and reduced spectral efficiency. To mitigate these effects, we explore adaptive signal processing, AI-driven equalization, and multi-band switching techniques. Additionally, our review offers a comprehensive assessment of the limitations of THz devices, including their operating temperatures, their detector sensitivity, the output power of light sources, and their modulation bandwidth constraints. Unlike previous reviews, which often provide a generalized discussion of THz sources, we analyze quantum cascade lasers (QCLs), difference frequency generation (DFG) techniques, and novel THz photonic mixers with respect to their feasibility for practical deployment in THz-FSO systems. This review serves as a valuable resource for researchers and engineers, providing quantitative analysis, mitigation strategies, and future research directions to overcome the existing limitations in THz-FSO hybrid networks.

## 2. Fundamentals of Terahertz Communication

### 2.1. The Terahertz Spectrum

The THz frequency range is approximately in the range from 0.1 THz to 10 THz and is between the microwave and optical frequency ranges [4,10]. It provides a bandwidth capability higher than 100 GHz, which is higher than the microwave system, which operates in a range from MHz up to a few GHZ only [16,20]. This tremendous transmission capacity, coupled with the ability to provide data rates of over 1 Tbps, makes the THz spectrum an important foundation for future wireless communications, and equally so for the envisaged 6G networks [21,22].

#### 2.1.1. High Bandwidth

The high bandwidth that can be achieved from the THz spectrum is one of the biggest strengths attributed to this spectrum. Microwave frequencies, which operate only in 1–10 GHz frequencies, cannot support the data intensity required by modern applications [23,24]. On the other hand, the THz spectrum has a bandwidth that is more than 100 GHz and up to 10 THz in some scenarios, with data rates of 1–10 Tbps. Such a bandwidth is essential for solutions like ultra-HD streaming, which requires data rates of at least 25 Mbps per stream, and 6G networks, which are expected to require at least 1 Tbps per user in peak scenarios [25].

**Figure 2:**
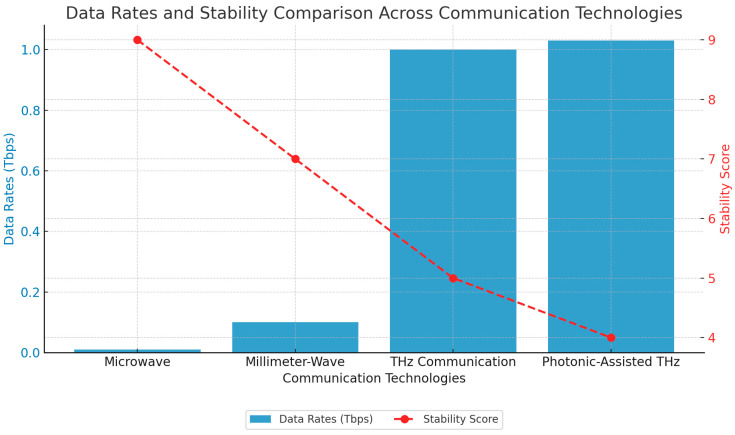
Data rates and stability comparison.

**Figure 3:**
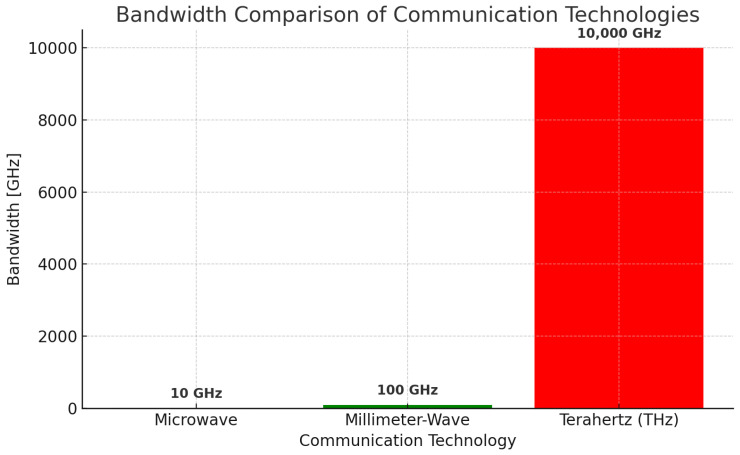
Bandwidth comparison of communication technologies.

Figure 2 shows a data rate and stability comparison. Furthermore, photonic-assisted technologies have been demonstrated to operate effectively in this bandwidth by attaining data rates of 1.03 Tbps per meter for the free-space optical link method in laboratory prototypes [26]. Metamaterial-based modulators and filters also play an important role in achieving great bandwidth characteristic improvements in usability in dense urban areas in terms of spectral efficiency [27,28]. Figure 3 illustrates the bandwidth capacities of different communication technologies, highlighting the significant increase in available bandwidth as we move from microwave to millimeter-wave (mmWave) and terahertz (THz) frequencies. The figure presents a comparative analysis of the bandwidth ranges for microwave (300 MHz–30 GHz), mmWave (30 GHz–300 GHz), and THz (300 GHz–10 THz) frequencies, demonstrating the exponential growth in spectral availability. Microwave technologies, which include conventional wireless and satellite communications, offer limited bandwidths (typically 1–10 GHz) but provide robust long-range capabilities. In contrast, mmWave communication, widely used in 5G backhaul and automotive radar, offers a 10–100 GHz bandwidth range, enabling high-speed short-range applications. The THz spectrum (300 GHz–10 THz), which is the focus for emerging 6G and ultra-high-speed wireless communication technologies, delivers from 100 GHz to several THz of bandwidth, making it an ideal candidate for next-generation holographic telepresence, inter-satellite links, and ultra-low latency applications. However, despite its vast bandwidth potential, THz communication faces challenges such as atmospheric attenuation, hardware limitations, and dispersion effects, which must be addressed to ensure practical deployment.

The channel capacity for a communication system can be determined using the Shannon–Hartley theorem, as shown below:

(1) $$C=B\cdot {log}_{2}\left(1+\mathrm{SNR}\right)$$

where C is the channel capacity in bits per second (bps), B is the bandwidth in Hz, and SNR is the signal-to-noise ratio [29]. In the context of THz systems, the bandwidth B can reach up to 1 THz in practical implementations, leading to unprecedented capacities exceeding 1 Tbps. However, in ideal scenarios, the theoretical bandwidth can extend up to 10 THz, although extreme absorption and device limitations significantly constrain the system’s usability at this rate [30].

For multi-antenna systems like MIMO, the channel capacity scales with the number of transmit ($N_{t}$) and receive ($N_{r}$) antennas:

(2) $$C_{\mathrm{MIMO}}=N_{t}\cdot N_{r}\cdot B\cdot {log}_{2}\left(1+\mathrm{SNR}\right)$$

Equation (2) models THz signal transmission, accounting for the power loss due to free-space path loss (FSPL) and atmospheric absorption. The received power (Pr) depends on the transmitted power (Pt), propagation distance (d), antenna gains (Gt, Gr), and wavelength (λ). As the distance increases, THz signals suffer from severe attenuation; thus, they require high-gain directional antennas and beamforming to achieve efficient transmission. Atmospheric absorption loss (L_atm_) is frequency-dependent, and THz-FSO systems experience significant losses above 500 GHz, making long-range THz communication challenging. FSPL ($L_{FSPL}$) further contributes to signal degradation, as higher frequencies experience greater power losses over long distances. To mitigate these effects, techniques such as multi-band adaptation, AI-driven power optimization, and low-loss material integration are essential for enhancing the stability and efficiency of THz links, particularly for 6G, inter-satellite links, and high-speed backhaul applications.

These equations highlight how the use of THz MIMO systems can further enhance the communication capacity of existing systems, making them ideal for high-demand applications like 6G and ultra-HD streaming. The THz spectrum is also shown to outdo traditional technologies in terms of bandwidth in Table 2, which depicts the data rates required for next-generation networks.

#### 2.1.2. Shorter Wavelengths

Terahertz frequencies with a range from 30 µm to 3mm can be designed and fabricated in a compact size and with a lighter weight due to the short wavelengths of the THz frequency range [31]. This is far different for microwave wavelengths, which range from 1 mm to 1 m (corresponding to 300 MHz–300 GHz), whereas millimeter wavelengths span 1 mm to 10 mm (30 GHz–300 GHz) and THz wavelengths are typically below 1 mm (300 GHz–10 THz), requiring larger, less compact parts [32]. For instance, antennas based on THz technology are capable of a physical dimension size of less than 1mm and, thus, can be incorporated directly on the chips of portable communication devices [17,33]. The recent development of integrated photonics as a large-scale system solutions allows us to directly emit THz signals with devices of less than 10 mm^2^ in size with up to 30% less power consumption than comparable systems [21,34]. Such elements are especially valuable in applications where size and mass are factors, such as in satellite communication. The relationship between the wavelength (λ) and frequency (f) in electromagnetic waves is given by the following equation:

(3) $$\lambda =\frac{c}{f}$$

Here, c = 3 × 10^8^ m/s is the speed of light. For THz frequencies ranging from 0.1 to 10 THz, λ falls in the range of 30 µm–3 mm, enabling compact and lightweight component designs for communication systems.

The diffraction-limited beamwidth for a system with an aperture diameter D is expressed as follows:

(4) $$\theta =\frac{1.22\lambda }{D}$$

THz communication, operating in the 300 GHz–10 THz range, provides an exceptionally high bandwidth for next-generation wireless networks. However, its high-frequency nature results in severe free-space path loss and atmospheric absorption, due particularly to molecular interactions with water vapor and oxygen, which drastically limit its long-distance transmission. These challenges necessitate the use of beamforming techniques, multi-band adaptive switching, and hybrid THz-FSO architectures to ensure reliable connectivity and optimize the system’s power efficiency and data rate performance in practical deployment scenarios. This makes THz systems capable of being targeted to a very small beam width and, therefore, of receiving little interference from adjacent beams; this type of system is ideal for use in urban and satellite applications.

**Figure 4:**
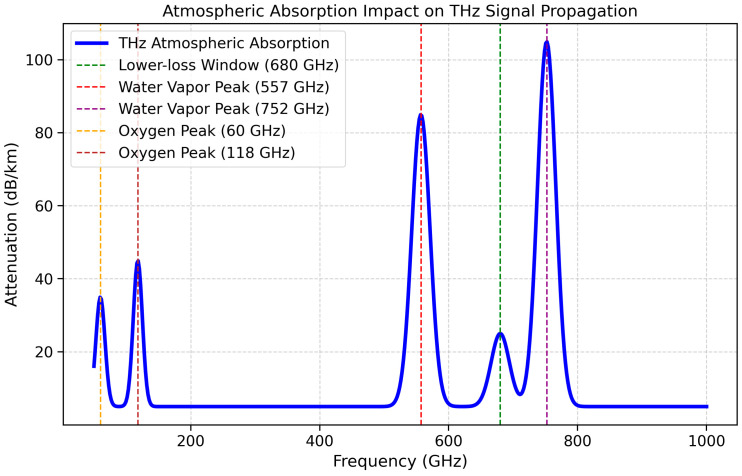
THz communication advancement spectrum.

The THz spectrum provided in Table 3 can make compact and lightweight components possible, transforming portable and on-chip technologies. Figure 4 provides an in-depth visualization of atmospheric absorption and its impact on THz signal propagation, emphasizing the severe attenuation that occurs as frequencies increase beyond 500 GHz. The figure plots attenuation (in dB/km) against frequency, illustrating key absorption peaks caused by molecular interactions with water vapor, oxygen, and other atmospheric gases. It highlights that THz waves experience dramatic signal loss beyond 500 GHz, where absorption can exceed 100 dB/km, severely restricting long-range transmission. However, the figure also identifies a lower-loss transmission window around 680 GHz, which presents a promising opportunity for optimized high-speed THz communication. The high-absorption regions correspond to the major spectral absorption lines of water vapor (~557 GHz, 752 GHz) and oxygen (~60 GHz, 118 GHz), making these frequencies unsuitable for long-distance communication. This visualization underscores the importance of applying frequency selection, adaptive multi-band switching, and AI-driven signal processing to mitigate the atmospheric losses. Additionally, hybrid THz-FSO systems and beamforming techniques could improve the feasibility of using THz links in real-world conditions.

#### 2.1.3. Higher Data Rates

The number of THz frequencies that are available is not restricted as in the other bands, which lends this frequency range to use for high-speed transmissions with a rate control of more than 1 Tbps, as this rate is feasible in specified settings [21,35,36]. In comparison, the typical rates for optical-based communication system range from 100 Gbps to 400 Gbps in commercial applications [37,38]. These promising features indicate that the THz spectrum can offer 10× higher data rates, making it applicable for such applications as 8K streaming video streams, which need 100 Mbps per stream, or inter-satellite links that will need 10–50 Gbps per channel [39,40].

Techniques in nanofabrication have also enhanced the spectral efficiency of THz systems by attaining 4 bits/Hz efficiencies in dense network conditions [22,41]. These advancements guarantee the readiness of THz technologies to cater to the increasing need for high-speed, low-delay communication in schools, urban centers, and all other places. The achievable data rate in a communication system is governed by the Shannon–Hartley theorem:

(5) $$R=B\cdot {log}_{2}\left(1+\frac{P}{N_{0}B}\right)$$

where R is the data rate in bits per second, P is the signal power in watts, $N_{0}$ is the noise power spectral density, and B is the bandwidth. For THz systems with B = 1 THz and high signal-to-noise ratios, R easily exceeds 1 Tbps.

In pulse-based THz communication, the data rate can also be approximated by the inverse of the pulse duration ($\tau_{p}$):

(6) $$R_{p}=\frac{1}{\tau_{p}}$$

Concerning ultra-short pulses with durations of 1 Gbps, the data rates can go up to 1 Tbps, which elicits confidence in the use of THz frequencies.

Table 4 highlights the data rates supported by THz (terahertz) communication technology. These data rates are essential for applications that depend on a high throughput, such as 6G networks, which require ultra-fast data transmission, and real-time analytics, which rely on the rapid processing of large datasets. Although the THz spectrum offers immense potential for a wide range of applications, it is not without its challenges. These difficulties must be addressed to fully harness its capabilities. The atmospheric attenuation, especially by water vapor, ranges between 0 and 20 dB per kilometer, particularly at specific bands, significantly reducing the range of this technology in especially wet environments [42]. These losses can only be overcome by using better materials like graphene base modulators, which have experimentally reduced the attenuation by about 40%. The economic viability of this technology remains a significant challenge, limiting its widespread adoption. Known THz sources, like quantum cascade lasers, are relatively expensive and cost from USD 10,000 to USD 50,000 per piece. Making THz technologies adaptable for commercial networks will also require the creation of cost-efficient manufacturing processes [43].

In conclusion, it can be stated that the THz spectrum is a frontier area with high potential for a major impact on the development of novel communication systems [44]. The changing needs of modern applications can be met using the high bandwidth, shorter wavelength, and ultrafast data rates provided by THz communication systems. Nevertheless, new investigations and developments are required to eliminate the above limitations and harness the capability of THz technologies in next-generation networks [37,45].

### 2.2. Advantages of THz in FSO Systems

Several aspects of using THz frequencies make them suitable for free-space optical communication systems, especially for next-generation applications. A significant advantage is the additional bandwidth provided by the THz range. Bandwidths in the THz range can hold data transfer rates of over 1 Tbps, which is far more than those of the microwave or optical communication systems [46]. This places THz communication in a very suitable place for use in data-hungry scenarios like 6G networks, HD streaming, and real-time analysis. Reports have shown that THz-based links support a data transmission capacity up to 4 Tbps for a short range [47].

Another equally important advantage is miniaturization, which is made possible by the fact that the THz frequency is shorter in wavelength. The range between 30 µm and 3 mm produces the possibility of developing compact antennas and fabricating elements that are suitable for integration into portable systems or on-chip [48]. Miniaturization lowers the general form factor of communication gear, making THz systems appealing for use in satellite communication equipment, UAV platforms, and wearable devices. The future development of nanofabrication could improve these compact designs further so that their energy utilization is efficient and their performance is equal to that of existing systems [18,49].

Specifically, the THz spectrum is also advantageous in terms of minimizing interference with currently running systems of communication [19,50,51]. Thanks to its low-frequency repetition rate, the THz band is not confused with the densely populated microwave and optical ranges and, therefore, has less signaling interference. This characteristic is extremely desirable in densely populated areas where signal interference is likely to affect the system’s operation. In addition, the implementation of THz signals with a narrow beamwidth improves the physical layer security by minimizing the chances of interception or eavesdropping [52,53].

## 3. Challenges in Terahertz-Based FSO Communication

Free-space optical (FSO) communication systems based on terahertz (THz) technology face several technical and environmental challenges that impact their deployment and reliability. One of the most critical challenges is atmospheric absorption, where water vapor and oxygen absorption cause severe attenuation at higher THz frequencies, exceeding 100 dB/km beyond 500 GHz, making long-distance communication impractical [1,2]. However, a lower-loss transmission window at 680 GHz offers a more viable option for THz-FSO links in specific applications [3]. The power loss due to atmospheric absorption follows the Beer–Lambert law, where the received power diminishes exponentially with distance and is influenced by environmental factors such as humidity, temperature, and pressure, leading to frequency-dependent attenuation [4,5]. Apart from absorption, line-of-sight (LOS) alignment is another major hurdle, as THz signals require precise beam alignment, and even slight misalignments can result in substantial power loss, which is further exacerbated by atmospheric turbulence and environmental vibrations [6,7]. Additionally, signal distortion due to scattering and refraction affects the efficiency of THz-FSO communication, for which the Kolmogorov turbulence model quantifies refractive index fluctuations and the Mie scattering theory explains airborne particle interference with THz waves [8,9]. Hardware constraints are also significant; existing quantum cascade lasers (QCLs) and photonic mixers both exhibit low power efficiency and require cryogenic cooling, increasing their operational costs and complexity [10,11]. Similarly, THz photodetectors suffer from low sensitivity and responsivity, limiting their large-scale deployment [12]. The development of high-efficiency THz components, including black phosphorus-based photodetectors and metamaterial-enhanced antennas, is crucial for improving communication systems’ performance, but their scalability remains a challenge due to high fabrication costs and precision engineering requirements [13]. To mitigate these challenges, software-defined networking (SDN) and multi-band adaptive frameworks are used to dynamically select the most efficient frequency bands for transmission [6]. Hybrid optical-THz-RF systems provide enhanced reliability by switching between THz, millimeter-wave, and optical frequencies, depending on the atmospheric conditions [14]. Moreover, artificial intelligence (AI)-driven network management is emerging as a promising approach to optimizing resource allocation, beam alignment, and error correction in real time [15]. Despite these issues, THz-FSO communication remains a promising solution for ultra-high-speed, secure, and low-latency networks, and addressing these limitations through frequency selection, low-loss material integration, the use of adaptive optics, and AI-driven optimizations will be critical for 6G, satellite, and high-speed terrestrial communication deployments [16,17].

### 3.1. Atmospheric Absorption

A significant constraint of THz-FSO communication is the amount of atmospheric absorption; this emanates from the interaction of the THz waves with water molecules in the atmosphere [16,54]. This results in exponential power losses, especially in areas of high humidity, which obstruct the signal, transmission range, and quality of the system [55,56]. The attenuation of THz waves due to atmospheric absorption can be mathematically modeled using the generalized form of the Beer–Lambert law, which accounts for varying frequencies and environmental parameters:

(7) $$A\left(f,d\right)=P_{0}\cdot e^{-\alpha \left(f\right)\cdot d}$$

where $A\left(f,d\right)$ is the remaining power at frequency f after the signal has travelled distance d, $P_{0}$ is the initial transmitted power, $\alpha \left(f\right)$ is the frequency-dependent attenuation coefficient (m^−1^), and d is the transmission distance (m).

The frequency-dependent attenuation coefficient $\alpha \left(f\right)$ is determined by the following:

(8) $$\alpha \left(f\right)=\sum_{i}\frac{S_{i}\cdot \gamma_{i}}{{\left(f-f_{i}\right)}^{2}+\gamma_{i}^{2}}$$

where $S_{i}$: the line strength of the i-th absorption line; $f_{i}$: the center frequency of the i-th absorption line; $\gamma_{i}$: the line width of the i-th absorption line (pressure and temperature dependent); f: the operating frequency (THz)

This model reflects the dependence of the attenuation on the specific absorption lines of atmospheric water vapor, highlighting the sensitivity of THz communication to environmental conditions.

#### 3.1.1. Mitigation Strategies

1. Photonic crystal fibers: hybrid photonic crystal fibers (PCFs) confine the optical field to low-loss regions, minimizing its interaction with water vapor and reducing (f) [7,8];
2. Frequency selection: operating at THz frequencies with minimal water vapor absorption peaks, such as 0.3 THz, significantly reduces the losses [8,15];
3. Adaptive beamforming: dynamically steering the beam to optimal transmission paths in response to weather variations helps mitigate attenuation [37,57,58];
4. Low-loss materials: advanced materials like metamaterials and dielectric polymers are engineered to enable efficient wave guiding and minimal absorption.

#### 3.1.2. Challenges in Implementation

High cost: The fabrication of low-loss PCFs and metamaterials is cost-intensive.

Bandwidth trade-offs: selecting specific frequencies reduces the available bandwidth for communication.

Complex integration: Adaptive systems require sophisticated algorithms and hardware for real-time operation.

#### 3.1.3. Advanced Modeling for Atmospheric Effects

The atmospheric attenuation also depends on additional environmental factors like the temperature (T) and pressure (P). The following advanced equation models these dependencies:

(9) $$\alpha \left(f,T,P\right)=k\cdot P\cdot {\left(\frac{300}{T}\right)}^{n}$$

where k: the proportionality constant (depends on gas type); P: the tmospheric pressure (Pa); T: the temperature (K); n: the temperature dependence factor

This formula allows for the dynamic adjustment of THz communication parameters based on real-time atmospheric conditions, improving the link performance.

**Figure 5:**
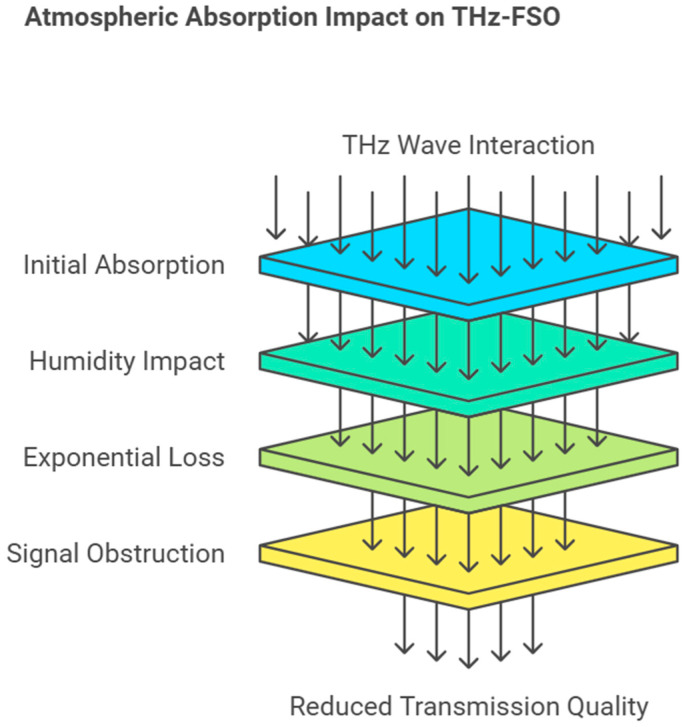
Atmospheric absorption.

Table 5 summarizes the challenges in THz communication due to the atmospheric absorption of water vapor, scattering, and environmental sensitivity. Possible solutions, including the use of photonic crystal fibers, low-loss materials, and adaptive beam steering, are presented, and their barriers, including their high costs and complex system integration, are discussed. The reduced transmission quality due to atmospheric absorption is depicted in Figure 5. 

### 3.2. Line-of-Sight (LOS) Alignment

Precise line-of-sight (LOS) alignment is fundamental, in THz-FSO communication systems, to maintain signal integrity and prevent beam divergence. Due to the short wavelengths of THz signals, even minor misalignments can result in significant power losses [50,53]. The received power ($P_{r}$) is typically calculated using the Friis transmission equation:

(10) $$P_{r}=P_{t}G_{t}G_{r}{\left(\frac{\lambda }{4\pi d}\right)}^{2}$$

where $P_{t}$: the transmitted power (W); $G_{t},G_{r}$: the gains of the transmitting and receiving antennas; λ: the wavelength (m); d: the distance between the transmitter and receiver (m).

The equation also indicates that the power received varies inversely with the square of the distance at a specific frequency; hence, high-frequency alignments require high accuracy. Additionally, the beam divergence (Θ) is a critical factor that depends on the aperture size and wavelength. A slight divergence angle ensures a focused energy transfer but increases the sensitivity of the signal to environmental perturbations such as turbulence and vibrations. Some mitigation strategies for this sensitivity are as follows:

- Adaptive beam steering: real-time adjustment of the beam direction to maintain alignment, even in dynamic scenarios;
- Vibration compensation systems: mechanisms that counteract environmental vibrations and platform instability;
- Advanced optics: the utilization of lens arrays and phased array antennas to improve the beam focusing and alignment precision.

Table 6 presents the major problems related to LOS alignment for THz-FSO communication systems, considering their causes, expected solutions, and corresponding demerits. Techniques like the use of adaptive optics, phased array antennas, and vibration compensation systems make a system more robust in dynamic environments.

### 3.3. Hardware Limitations

THz communication systems have strong hardware restrictions, which is one of the main barriers to THz development and realization. Present THz gadgets, including QCLs and photodetectors, have low efficiency, low power, and a high fabrication cost. These challenges also make it difficult to realize large-scale and low-cost THz communication systems [59].

THz communication depends on the technology of quantum cascade lasers, which produce less than 10% efficiency on the best of occasions, to provide adequate power for long-range transmission [12,13,60]. QCL systems face added challenges due to the need for cryogenic cooling to drive current and laser operations, which considerably raise their operational costs and complexity. To overcome these problems, new high-efficiency semiconductor materials and design configurations like distributed feedback structures are in the pipeline [61].

Photodetectors, another critical component of every system, are also characterized by sensitivity and responsivity problems at THz frequencies. Recent advancements in new concepts such as black phosphorus-based materials are expected to improve the performance of detectors and reduce their production costs. The challenge of scalability and cost is a major issue in the development of these materials. In the same vein, unitraveling-carrier photodiodes (UTC-PDs) [10], which are designed to generate high-power THz signals, are only adequate in terms of their power conversion efficiency.

In addition to the above, there are design challenges that arise with the antenna, thanks to the short wavelengths exhibited by THz signals [62,63]. Miniature antennas require critical assembly procedures which contribute to the cost and development of THz instruments. Currently, there appear to be ongoing attempts to combine metamaterials with phased array technology to improve antenna system performance, but this solution has not come of age for volume production yet. A comparative summary of key THz device characteristics, including their operating temperature, detector sensitivity, output power, and modulation bandwidth, is presented in Table 7.

The above table presents the major hardware issues in THz communication: low efficiency, high cost, and scalability. There are limitations to existing LiDAR systems, and proposed solutions, like enhanced materials and optimized photodiode designs, are offered to overcome these limitations, albeit at the cost of increased prices and manufacturing challenges.

### 3.4. Signal Distortion

In THz communication, signal degradation is caused by turbulence and scattering in the air, and significantly degrades the signal [64,65]. These distortions are quantified using metrics such as the scintillation index ($S_{I}$), a measure of the intensity of fluctuations caused by turbulence:

(11) $$S_{I}=\frac{\langle I^{2}\rangle -\langle I_{int}\rangle^{2}}{\langle I_{int}\rangle^{2}}$$

where $\left\langle I_{int}\right\rangle$: the average signal intensity. $\left\langle I^{2}\right\rangle$: The mean square signal intensity.

Higher $S_{I}$ values correspond to more substantial turbulence, which leads to increased signal degradation. Atmospheric turbulence can also be described using the Kolmogorov turbulence model [22,38], which relates the refractive index structure parameter ($C_{n}^{2}$) to the turbulence strength:

(12) $$\Phi_{n}\left(k\right)=0.033C_{n}^{2}k^{-11/3}$$

where $\Phi_{n}\left(k\right)$: the power spectral density of refractive index fluctuations. $C_{n}^{2}$: the refractive index structure parameter ($m^{-2/3}$). k: the wavenumber ($m^{-1}$).

Additionally, signal distortion due to scattering can be modeled using the Mie scattering equation for spherical particles:

(13) $$Q_{s}=\frac{2}{x^{2}}\sum_{n=1}^{\infty }\left(2n+1\right)\left({\left|a_{n}\right|}^{2}+{\left|b_{n}\right|}^{2}\right)$$

where $Q_{s}$: the scattering efficiency. $x=\frac{2\pi r}{\lambda }$: the size parameter (ratio of particle size r to wavelength λ). $a_{n},b_{n}$: the scattering coefficients for spherical harmonics.

These equations provide a mathematical framework to understand and quantify the impact of turbulence and scattering on THz communication. The mitigation strategies for these impacts include the following:

- Adaptive equalization: machine learning-based adaptive equalizers dynamically compensate for distortions;
- Beam shaping: adjusting beam profiles to counteract turbulence effects improves signal stability;
- Error-correction codes: advanced error-correction techniques reduce the impact of distortion on the data accuracy [66].
- Real-time sensing: using real-time environmental monitoring to optimize transmission paths;
- Polarization techniques: exploiting polarization diversity reduces the sensitivity to turbulence-induced scattering.

The key mathematical issues identified in Table 8 concern the power of THz signals, the relationship between turbulence and scattering, and potential solutions for reducing THz signal distortion. The problems are well solved by techniques like adaptive equalization, beam shaping, and real-time sensing, but at the cost of increased computational complexity, costs, and energy.

### 3.5. Atmospheric Dispersion and Its Impact on THz-FSO Communication

One of the significant challenges in THz-FSO communication that has often been overlooked is atmospheric dispersion, which further limits the usable instantaneous bandwidth and degrades high-data-rate transmissions. Dispersion arises due to the frequency-dependent propagation delay, which distorts signal waveforms and leads to pulse broadening and inter-symbol interference (ISI) [66]. The primary cause of dispersion in the THz band is the variation in the refractive index of the atmosphere due to molecular interactions with humidity, temperature fluctuations, and other environmental factors. This effect is particularly severe at higher THz frequencies, where the phase velocity varies across different spectral components, causing severe signal distortion.

As the THz signal propagates through the free-space optical channel, it undergoes group velocity dispersion (GVD), which causes different frequency components to travel at different speeds, leading to the temporal spreading of the signal pulse [67]. This spreading effect reduces spectral efficiency, increases bit error rates (BERs), and severely impacts high-speed data transmission in THz-FSO systems. The combined impact of dispersion and atmospheric absorption further exacerbates the transmission loss, limiting the feasibility of long-range THz-FSO communication, particularly beyond 500 GHz, where the attenuation surpasses 100 dB/km [68]. However, research suggests that a relatively lower-loss transmission window exists at 680 GHz, which may provide a practical frequency band for optimized high-data-rate THz-FSO links [55].

#### Mitigation Strategies for Atmospheric Dispersion

Several techniques have been proposed to counteract the effects of dispersion in THz-FSO communication. Adaptive modulation and equalization techniques, including machine learning-based adaptive equalizers, can be deployed to compensate for phase and frequency dispersion, ensuring real-time signal restoration [69]. Additionally, pre-compensation techniques at the transmitter can mitigate dispersion effects by shaping signals to counteract the anticipated spectral broadening along the THz-FSO propagation path [70]. Frequency selection also plays a crucial role in mitigating dispersion; for instance, choosing 680 GHz transmission windows can significantly reduce the impact of frequency-dependent propagation delays. Moreover, optical filtering techniques, particularly photonic-assisted dispersion compensation, can be employed to dynamically correct spectral broadening effects, improving the system’s performance [71]. The integration of AI-driven network adaptation can further enhance THz-FSO communication by dynamically adjusting transmission parameters to optimize the spectral efficiency under varying atmospheric conditions [1].

Despite these challenges, THz-FSO systems remain promising for use in ultra-high-speed, secure, and low-latency communication networks, provided that the dispersion effects are effectively mitigated. Future research should focus on the co-design of low-loss waveguides, optical dispersion compensation filters, and AI-driven adaptive beam alignment, ensuring that THz-FSO technology can meet the demands of next-generation networks, including 6G, inter-satellite links, and high-speed terrestrial communication [72].

### 3.6. Manufacturing Challenges in THz Communication

There are several manufacturing challenges that limit the large-scale deployment of THz communication systems. Unlike microwave and millimeter-wave technologies, THz systems require high-precision fabrication, specialized materials, and advanced device integration to achieve efficient signal generation, transmission, and detection.

#### 3.6.1. High-Precision Fabrication and Nanometer-Scale Tolerances

THz components, such as waveguides, antennas, and resonators, demand nanometer-scale precision due to their extremely short wavelengths (sub-millimeter range). Conventional microfabrication techniques, such as photolithography and electron beam lithography, face difficulties in maintaining the necessary precision and repeatability at THz frequencies. Moreover, surface roughness and material imperfections introduce significant scattering losses, degrading the overall system performance. The transition from silicon-based fabrication to novel nanomaterials like graphene, black phosphorus, and topological insulators could mitigate some of these issues, but introduces new manufacturing complexities and cost concerns [10].

#### 3.6.2. THz Transmitter and Receiver Limitations

Efficiently generating and detecting THz signals remains one of the biggest hurdles in THz communication. Current THz sources, such as photonic-based quantum cascade lasers (QCLs) and Schottky diode-based mixers, exhibit low power conversion efficiency and require cryogenic cooling to achieve optimal performance. Similarly, THz detectors, including bolometers and high-electron-mobility transistors (HEMTs), lack the sensitivity and dynamic range needed for high-speed communication. Achieving room-temperature operation with high efficiency remains a key manufacturing challenge, requiring innovations in semiconductor processing and metamaterial engineering [40].

#### 3.6.3. Material Constraints and High Absorption Loss

The traditional metallic and dielectric materials used in RF and optical components exhibit high losses at THz frequencies, particularly due to free-electron absorption and polarization effects. The selection of low-loss materials, such as high-resistivity silicon, indium phosphide (InP), gallium arsenide (GaAs), and 2D materials like graphene, remains an active research area. However, the scalability and fabrication costs of these materials pose major obstacles for commercial THz communication devices [57].

#### 3.6.4. Packaging and Integration of THz Systems

THz components must be seamlessly integrated into compact, high-performance systems while ensuring thermal stability and electromagnetic shielding. Unlike traditional RF and optical systems, THz circuits require the precise alignment of photonic and electronic elements, which significantly increases their manufacturing complexity and costs. The transition toward the monolithic integration of THz photonics and electronics (e.g., THz CMOS and III-V semiconductors) could address these challenges but demands significant advancements in hybrid chip fabrication and interconnect technology [73].

#### 3.6.5. Scalability and Cost Barriers

The high manufacturing costs associated with THz technology have prevented its widespread adoption in commercial applications. Unlike the use of millimeter-wave technology in 5G networks, which benefits from mass production, THz components require specialized fabrication processes and exotic materials, making them significantly more expensive. Standardizing wafer-scale fabrication techniques for THz systems, similar to the approach used in silicon photonics, are necessary to drive down costs and enable the mass production of these systems [55].

## 4. Enabling Technologies for THz-FSO Communication

**Figure 6:**
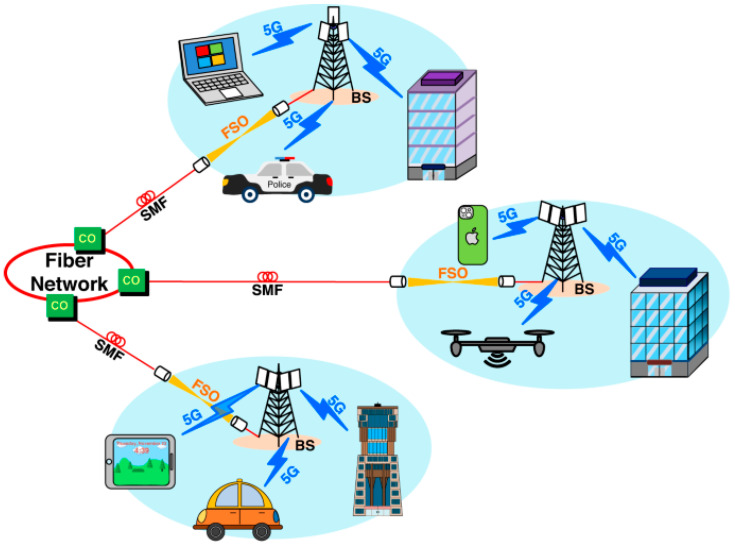
Enabling technologies for THz-FSO communication.

Recent discoveries in material science, device engineering, and chip-to-fiber photonics integration have made terahertz (THz)-based free-space optical (FSO) communication systems more efficient [63,67]. Enabling technologies are discussed in this section as major factors that are shaping developments in the study area, represented in Figure 6. 

Unlike traditional RF and optical communication, where direct modulation of the source is commonly used, many THz sources—such as quantum cascade lasers (QCLs) and photomixers—cannot be efficiently modulated using a pumping current due to slow carrier dynamics and thermal constraints [32]. In such cases, external THz modulators become essential for encoding information onto THz carrier waves. Depending on their operating principle, THz modulators can be classified into electronic, optical, and hybrid modulators [34]. Based on field-effect transistors (FETs) and Schottky diodes, electronic modulators enable high-speed amplitude and phase modulation, but suffer from limited efficiency at higher THz frequencies. Optical THz modulators, such as those using plasmonic metasurfaces and electro-optic materials (e.g., lithium niobate, graphene, and black phosphorus), provide ultra-fast modulation speeds and broadband tunability, making them more suitable for future THz communication networks [45]. Hybrid approaches, such as optically controlled modulators, integrate photonic and electronic components to achieve higher efficiency and faster response times. For efficient THz-FSO (free-space optical) communication, modulators based on graphene, metamaterials, and phase-change materials are being developed to achieve dynamic tunability and higher modulation depths [57]. Metasurface-based THz modulators offer enhanced beam steering, polarization control, and spatial multiplexing, making them ideal for use in adaptive THz communication architectures. Integrating machine learning-based adaptive modulation schemes is another promising approach to optimizing the transmission efficiency of THz-FSO systems under varying atmospheric conditions. Given the critical role of THz modulators in enabling high-speed data transmission, future research should focus on low-loss, energy-efficient, and scalable modulator technologies that can be seamlessly integrated into 6G-and-beyond networks [68].

### 4.1. Advanced Materials

Recent innovations in material thickness have acted as the driving foundation of the fabrication of THz-FSO group communication systems. These materials facilitate critical operations like high-speed signal processing, enhanced sensitivity, and low power consumption, which form the framework for building efficient THz-FSO networks [55,68,69].

Graphene and 2D materials: THz-FSO systems have been enriched with THz waves’ excellent features, which are embodied by graphene or other 2D materials [43]. The client on-chip THz measurement capability of graphene is attributed to its high carrier mobility and ultra-fast carrier dynamics for THz signal modulation and detection. Its tunable conductivity, as governed by the Drude model, allows for adaptive device functionality, enhancing the bandwidth and energy efficiency of THz devices:

(14) $$\sigma \left(\omega \right)=\frac{e^{2}}{\pi \hbar }\frac{\mu }{1+{\left(\omega \tau \right)}^{2}}$$

Graphene devices exhibit harmonious integration with compact and flexible designs, making them suitable for portable THz-FSO systems. However, a problem exists regarding the scalability of graphene production and its compatibility with present-day device structures. There is also the development of problems such as inadequate electrical conductivity and the need for more attractive, lighter, or cheaper materials that can be formed through chemical vapor deposition (CVD).

Metamaterials: THz metamaterials with engineered nanoscale structures feature promising capabilities in terms of the phase, amplitude, polarization, direction, and frequency of THz waves [70]. They are indispensable in controlling the beam, wavefront, and polarization in THz-FSO systems, modeling refractive indices that are negative and tunable. The relationship between reflectance, transmittance, and absorption can be represented as follows:

(15) R+T=1−A

These materials have been used to fabricate high-accuracy THz lenses and waveguides with low loss and superior positioning accuracy. However, the high cost and difficulty of mass producing metamaterials are still the main issues. Subsequent innovations in optimizing the nanoparticle have also boosted the effectiveness of metamaterial devices, cutting down on light loss and representing a general upgrade of the host systems.

Nanoparticles and hybrid materials: the incorporation of nanoparticles into photonic fibers has been demonstrated to minimize the dispersion and light loss in THz-FSO systems [35,71]. Optically dense nanoparticles can help improve spot size localization and can permit changes on the wavefront within a fly’s time. In the same way, new compounds, including ITO/SeO_2_, are characterized by enhanced optical modulation characteristics that can increase the information transfer rate and ensure the process’s reliability.

$ITO/SeO_{2}$ interfaces: The material that has most recently been found suitable for use in the fabrication of optical modulators for THz-FSO systems is indium tin oxide (ITO)-selenium dioxide $\left({SeO}_{2}\right)$. These interfaces cause this link to experience losses and, at the same time, allow for high modulation depths [1,72]. Pertaining to both designs and simulations, their modulation stability is considered as a strength, while their non-linearity effects are minimal. Nevertheless, their potential to obtain a uniform material property over large areas is still a challenge.

Table 9 presents details on the constituents and use of high-performance THz-FSO system enablers such as graphene, metamaterials, and hybrid nanoparticles. These materials offer specific approaches to problems associated with dispersion, light loss, and wavefront control while exposing the more minor problems that are still left to be solved, such as affordable fabrication and integration.

### 4.2. Integrated Photonic Devices

Incorporating photonic devices within THz-FSO systems offers great potential by advancing their overall communication in terms of their data rate, latency, and energy consumption. These devices play significant functions in data flow, real-time computation, and system extensibility [2,10,42].

Quasi-optic modules: quasi-optic modules for the 60–180 GHz range have been widely applied to terahertz frequency-selective optics systems. These modules employ Avangel photonic integration that increases the signal bandwidth, thus leading to higher data rates [76]. Their quasi-optic structures improve the system’s beam pointing and allow efficient wireless-to-optical interconversion. The evolution of new versatile quasi-optic receivers has also made their deployment compatible with future-generation communication systems. However, perfectly matching the alignment across particularly differing environmental conditions is proving to be rather difficult.

On-chip photonic circuits: programmable pulse processing functions have arisen from on-chip photonic circuits that consist of cascaded Mach–Zehnder interferometers (MZIs) and micro-ring resonators (MRRs) [13,77]. MZIs employ phase modulation to achieve dynamic signal control, with phase shifts governed by

(16) $$\Delta \phi =\frac{2\pi \Delta L}{\lambda }$$

where Δϕ: the phase difference. ΔL: the path length difference. λ: the wavelength.

These circuits are critical to realizing THz frequency data processing in real-time. Their use enables flexibility in how the system responds to signals, although they require accurate fabrication and subsequent tuning to the appropriate parameters. There are discussions of using advanced manufacturing technologies, including lithography, to optimize the reproducibility of these devices.

Cascaded MIMO equalizers: Cascaded MIMO equalizers, especially, are used to perform data integration for fiber-THz wireless systems. These devices allow straightforward switching between the optical mode and THz mode, making the computational bandwidth very high and exhibiting low latency [76]. Since MIMO equalizers help to minimize different distortions that resulting from the multipath, they trigger signal characteristics and allow for the reliability of a given system. However, their deployment demands enhanced energy utilization, which is a hurdle in resource-poor settings.

In Table 10, we defined the functional aspects of and developments regarding integrated photonic devices in THz-FSO systems. From the quasi-optic modules to superior waveguides, all these devices serve to improve data throughput rates and signal readability. However, issues like the accuracy of fabrication, energy issues, the loss of material, etc., call for more study and development.

### 4.3. High-Power THz Sources

Moreover, the production of high-power THz signals is crucial, especially for enhancing the data rate and for the effective realization of THz-FSO systems in long-distance communication. Significantly improving the performance of these systems’ QCLs, photonic mixers and FELs have recently undergone several improvements [41,66,73].

Quantum cascade lasers (QCLs): QCLs are among the smallest and most efficient sources of THz radiation, and they employ broadband electron transitions between multiple quantum wells to produce high-power coherent THz waves [13,73,78]. Both designs offer the potential for adjusting emission frequencies with a high degree of accuracy and are versatile. The output power ($P_{out}$) of QCLs is influenced by their quantum efficiency (η) and operating conditions:

(17) $$P_{\mathrm{out}}=\eta \cdot \frac{VI}{\lambda }$$

where $P_{\mathrm{out}}$: the output power. η: the quantum efficiency. V: the voltage. I: the current. λ: the wavelength.

THz QCLs have excellent features that lend themselves to incorporation in compact THz systems, especially in long-range communication systems. However, they are not very efficient in generating large amounts of power due to requiring expensive cooling mechanisms to be embedded in them. Constant research is being undertaken to explore the use of other lighter and high-strength materials, and to find better thermal management systems.

Quantum cascade lasers (QCLs) are among the most promising THz light sources due to their ability to operate in the mid-infrared and THz spectra with high power efficiency. However, unlike edge-emitting semiconductor lasers that operate in the near-infrared range, the direct modulation of QCLs using a pumping current remains a significant challenge due to carrier lifetime limitations and thermal effects. Traditional near-infrared edge-emitting lasers can achieve high-speed direct modulation up to several GHz by manipulating carrier injection dynamics and photon lifetimes [79]. In contrast, THz QCLs experience longer carrier lifetimes and slower electron transitions, restricting their modulation bandwidth to much lower values. This limitation suggests that external THz modulators or hybrid photonic–electronic modulation techniques are necessary to optimize THz signal encoding for high-speed wireless communication. THz photonic mixers are widely used for frequency conversion and the generation of high-purity THz signals, and typically rely on optical heterodyning or nonlinear difference-frequency generation (DFG) techniques. While this review discusses various frequency mixing approaches, it previously overlooked the novel integration of DFG-based THz sources within QCLs. Recent advancements in DFG-QCLs demonstrate that these devices can achieve high spectral purity, tunability in the 1–6 THz range, and room-temperature operation, making them suitable for high-speed THz wireless networks [80]. Unlike conventional THz sources, which require external nonlinear crystals, DFG-QCLs incorporate nonlinear gain media within the laser cavity, enabling the fabrication of compact, monolithic THz emitters with superior performance. These properties make DFG-QCLs highly valuable for future 6G communication, spectroscopy, and secure transmission applications, as they address key challenges in THz signal generation and high-frequency transmission.

Photonic mixers: Photonic mixers work on difference frequency generation that effectively generates THz radiation out of optical signals. These mixers are recognized for their potential to produce broadband THz signals, which is important to support high-count data transmission in FSO systems [81,82]. The wide operating bandwidths offered by photonic mixers make them suitable for use where multi-channel data links are necessary; for instance, in spectroscopy and imaging.

The combination of photonic mixers with FSO systems has also improved their spectral efficiency and signal distortion at different atmospheres. Nevertheless, phase coherence remains a technical challenge even when using these UGMs, especially due to variability in the communication process.

Free-electron lasers (FELs): THz radiation is generated by the interaction of high-energy electron beams with periodic magnetic fields, which can produce highly coherent and efficient energy FELs. These systems are entirely appropriate for scientific research as well as other applications that must utilize very high levels of power, including material analysis or other imaging processes [10,22,28,73]. Due to their high output power and tunability, FELs are appropriate sources of radiation for use in high-precision applications.

However, FELs are inherently bulky, are costly to operate, and require complex maintenance, making them unsuitable for commercial THz-FSO applications. Current research on compact FEL designs intends to overcome these limitations, and recent progress has been made in both their compactness and cost reduction.

A comparison of the compactness, power output, and operating efficiency of various THz sources is depicted in Table 11. These technologies are still being further developed to optimize these parameters to fulfill multiple demands of THz-FSO systems.

To improve the efficiency of high-power THz sources, efforts are being made toward the identification of integrated systems that include both the characteristics of QCLs and those of photonic mixers [83,84]. Furthermore, the existing photonic devices are gradually being upgraded to use the latest and best materials like graphene in a bid to increase their wattage efficiency and limit heat production.

### 4.4. Adaptive Optics

Adaptive optics (AO) systems are of significant importance in the control of the atmospheric distortions that affect THz-FSO communication. These systems adaptively counteract wavefront errors induced by turbulence to support reliable communication links [64,85].

Wavefront correction: wavefront correction guarantees signal quality by adjusting for distortions that occur due to the atmosphere in real-time [61,85,86]. The effectiveness of AO systems is often quantified using the Strehl ratio (S):

(18) $$S=e^{-\sigma^{2}}$$

where S: the Strehl ratio, representing the optical quality. $\sigma^{2}$: the variance of wavefront errors.

These Strehl ratios characterize the optical quality, which makes the THz-FSO signal more faithful. Distortions due to turbulence substantially reduce the quality of the transmitted wave over long distances, which makes wavefront correction a key component of AO systems.

Real-time AO systems employ high-speed actuators and wavefront sensors to control the transmitted beam’s phase in real time [65,87]. They also play an important role in these systems as deformable mirrors, and Shack–Hartmann wavefront sensors are employed for the correction of distortions in real-time [25,88]. The performance of real-time systems is governed by their response time (T):

(19) $$T=\frac{\tau +\Delta t}{R}$$

where τ: the time constant of the actuator. Δt: the sensor delay. R: the control loop bandwidth.

Optimal AO processes are characterized by low-sensitivity delay, which makes it possible to respond effectively to fluctuations in the surrounding environment. This feature is especially useful in situations characterized by turbulence or dynamism within the organization’s operating environment [89,90].

Phase-only spatial light modulators: these are utilized in AO systems to provide high-resolution wave-front corrections without altering the amplitude of the passed-through signal. These devices can operate with a sepcific level of phase control to successfully correct the distortions in the atmosphere. The phase modulation (ϕ) introduced by an SLM is described as follows:

(20) $$\phi =\frac{2\pi d\Delta n}{\lambda }$$

where d: the thickness of the modulator layer. Δn: the refractive index change. λ: the wavelength of the light.

SLMs have high spatial resolution and are known to be suitable for application in correcting phase distortions that exist in a THz-FSO system.

Table 12 compares adaptive optical technologies with their specific strengths and weaknesses. Wavefront correction increases the quality of the signal but reduces the throughput, and algorithms and real-time AO systems that can adaptably correct signals over large distances also reduce the speed of the hardware. Phase-only SLMs provide accurate phase control on the sub-phase level but cannot perform rapid modulation, making them suitable for the wavefront correction of turbulence-induced spatial phase distortions.

### 4.5. Signal Processing Algorithms

Algorithms used in signal processing contribute significantly to overcoming obstacles that arise due to the fluctuating channel conditions in THz-FSO systems. These algorithms should improve link availability, data throughput, and latency, which are fundamental to efficient system operation.

Machine learning for signal reconstruction: the current state-of-the-art deep learning algorithms for untarring distorted THz signals include convolutional neural networks (CNNs) [55,74,94]. These models involve pattern recognition and the utilization of recorded signatures from which the models conclude the details of real-time environments to predict and compensate for distortions of the signals in real time. These implementations effectively enhance the signal-to-noise ratio and ensure data efficiency for even relatively unstable channels. However, concocting corrected signals for high-error-rate channels through the use of generative adversarial networks (GANs) is promising [95,96].

Beam alignment optimization: RL is used in the present analysis to dynamically schedule the alignment of THz beams through reinforcement-learning-based algorithms. These methods guarantee little or no pointing direction errors and improve energy utilization due to the constant changes in the pointing direction of the transmitter. The algorithms determine the conditions in the environment and act depending on the amount of turbulence when less power is used. These optimization methods are generally validated via Monte Carlo simulations, which compare the performance of the method under various circumstances.

Error correction codes [85,87]: Low-density parity-check (LDPC) codes and turbo codes, for example, are an essential component of reducing data loss in noisy conditions [2,6,7]. In this case, LDPC codes are especially efficient because they provide near-capacity performance along with reduced complexity. The effectiveness of these codes is measured by the bit error rate (BER), which is calculated as follows:

(21) $$BER=Q\left(\sqrt{\frac{2E_{b}}{N_{0}}}\right)$$

where BER: the bit error rate. Q: the Q-function, representing the tail probability of the standard normal distribution. $E_{b}$: the energy per bit. $N_{0}$: the noise power spectral density.

LDPC codes are known to offer good performance in high-interference channels and, therefore, are recommended for use in correcting errors in THz-FSO systems.

Input: the received signal ($S_{r}$); channel state information (CSI) output: the corrected signal ($S_{c}$) develop a deep learning model for reconstruction and establishing the estimations of channel impairments can be done by using CSI. Reinforcement learning-based beam alignment optimization can also be done in this way. Optimizing signal equalization and noise removal can be done as well. The received signal can be decoded using LDPC or turbo codes.

The algorithm discussed above combines the use of artificial intelligence and conventional signal processing to deliver the best result for THz-FSO links. It is possible to optimize data communication, since these techniques guarantee that information can be reliably sent across harsh environments.

## 5. Applications of THz-FSO Communication

The innovation in the field of THz-FSO communication has transformed many industries by providing high data rates, low latency, and high security. The following examples include space communication and integration in seventh-generation networking, like 6G.

### 5.1. Space Communications

The THz-FSO system works well with space applications because effect of the high frequency is reduced by cosmic noise. These systems meet today’s increasing bandwidth requirements for Intersat and satellite-earth links [97,98]. The high data rates offered by the THz-FSO system are of paramount importance to real-time data transmission, especially for space research, including space exploration, earth observation, and deep space communication [40].

In THz-FSO communication systems in space, electromagnetic interference would not be a factor because THz waves do not interfere with instruments on board a spacecraft, making them a reliable means of transferring sensitive data. THz devices are compact and can achieve data rates of up to gigabits; therefore, THz payload designs in satellites are well-optimized for this application. Moreover, the modern trends in narrow-bandgap material diversification have increased THz-transmitters’ effectiveness in terms of their power consumption and the effective transmission distance between space vehicles [41,75]. The THz-FSO systems offer reliable communication links in atmospheric re-entry situations, as plasma-induced sudden signal losses frequently occur in radio-frequency systems. These features give significant importance to THz-FSO communication in the differentiation of modern space technology.

### 5.2. 6G and Beyond

THz-FSO systems are primary candidates for augmenting 6G networks, as they represent a shift in the paradigm of telecommunication due to providing ultra-low latency, a high-capacity, and low energy consumption. Modern-day applications for which THz-FSO systems are essential in terms of data and latency include holographic telepresence, autonomous vehicle coordination, and industrial automation [43,45,57,99].

THz-FSO systems, in the context of 6G networks, provide the ULLC owing to the high spectral efficiency of the optical link. This makes them suitable for use in applications such as real-time augmented and virtual reality applications as well as in smart city applications. In addition, the THz-FSO system, which supports multi-6G device connectivity with lesser interference, complements the vision of 6G [57,58].

One technological innovation in this area is the utilization of terahertz polymer vortex photonic crystal fibers that guarantee the stable transmission of orbital angular modes. Introducing these fibers enhances the capability and reliability of 6G backhaul networks and will create grounds for new ways of incorporating THz-FSO links [100]. Furthermore, the utilization of multifunctional integrated chips for the THz-FSO link also minimizes the power consumption of the system and, therefore, minimizes the operational costs, leading to faster growth of these systems in future networks [8,42].

As the need for a higher data rate and more connectivity increases, THz-FSO communication will continue to spearhead technological advancement in telecommunication.

### 5.3. Secure Communication

The operating frequencies of these systems are inherently of a narrow beam width; hence, the signal leakage is very low, which is effective for military and financial communication. The small dispersion of THz beams also guarantees that all signals are channeled to proceed on a certain path, thus enhancing the security of the data that are being transmitted. However, new approaches involving sophisticated single-phaser detection methods also improve the security of THz-based systems by allowing the accurate identification of the occurrence of eavesdropping on the system [6,7,8].

THz-FSO communication systems are also immune to jamming and interferences since they operate in short wavelengths and are highly directive; hence, they can be employed in military communication networks. Optical feeder link models have shown better predictions in terms of accuracy and reliability and are important in bidirectional links where the applications of portions of the link may require high levels of security [3,4,6,9]. In addition, these systems utilize quantum encryption schemas to enhance the privacy of the user as well as to mitigate possible cyber threats [25,33,42].

In financial sectors, THz communications systems allow link networks to transfer high-value data between financial institutions and prevent possible attacks on data. The application of hollowness in fiber systems for THz transmission has allowed the highest data rate observed to date, as these systems are free from latency and immune to security threats, which the current financial networks require. Furthermore, abstraction has also improved the performance and stability of waveguide-based systems in coupled THz communication systems, guaranteeing their relevance for highly sensitive uses [37,45].

The next step in developing these systems is integrating terahertz-wave generation using periodically inverted gallium arsenide, which has increased the reliability and security of these systems, enabling their use in creating secure communication systems. For instance, photonic crystal fibers have been used to identify instances of violation of the signal integrity within holistic communication lines [36,101]. THz-FSO systems are expected to maintain a steady increase in demand for use in new communication solutions, where significant security-related challenges have emerged in recent years and where innovative technologies are constantly being sought for both civilian and military applications [78,100].

## 6. Synergy with Hybrid Communication Systems

**Figure 7:**
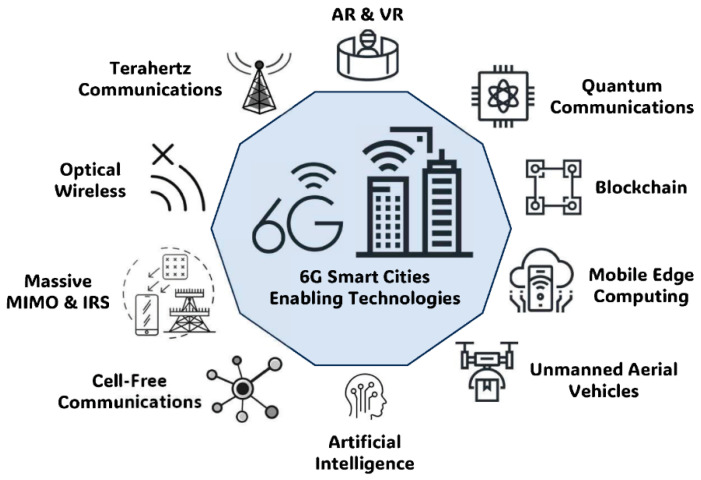
Synergy with hybrid communication systems.

Combining terahertz-bandwidth FSO with other communication modalities provides a sound approach to overcome FSO’s weaknesses. THz-FSO can be integrated with microwave or millimeter-wave systems, making an increased reliability, range, and system efficiency possible, as depicted in Figure 7.

### 6.1. Hybrid Optical-RF Systems

Optical radio frequency (RF) combines optical and RF links to enable a smooth transfer of all kinds of communication despite the environment. For example, optical link transmissions offer high-capacity communication in good weather. In contrast, an RF link assures connectivity in situations where there is fogginess or rainy conditions, which can affect the transmission of optical signals. This complementary approach improves the system’s reliability and excludes communication interruptions [25,102].

A typical example of this integration synergy is the use of RF-optical combined systems adopted for 5G and beyond networks that are enhanced by beam-steering in even more dynamic conditions. Interference and the throughput are minimized with regard to the positioning of the beam in relation to other beams [25,103].

### 6.2. Multi-Band Adaptive Systems

Flexible transceiver schemes select communication links at microwave, millimeter-wave, and terahertz (THz) bands according to the prevailing conditions and availability of resources. Such systems take the use of each range of frequencies to balance the system’s range, data rate, and reliable links [25,36]. For instance, although THz bands can provide up to UHDR, these are less robust and can be attenuated; hence, the system has to switch to microwave or millimeter-wave bands during unfavorable conditions.

Table 13 highlights the comparative advantages and limitations of these bands for hybrid systems.

### 6.3. Software-Defined Networking (SDN)

SDN is the key enabler in orchestrating hybrid communication networks because it acts as a compressor that addresses how critical resources and routing are allocated in real time. SDN controllers can autonomously change parameters in the selected links to assure optimality and manage bandwidth or frequency bands based on the current conditions [24,45].

Also, the integration of SDN with THz-FSO systems can optimize the resources that are available in multiband systems. For instance, applications such as HTP or autonomous vehicle networks rely on minimized delay and boosted data transfer rates that result from routing based on dynamic SDN.

## 7. Future Directions

Despite their current state of development, mainly in point-to-point connections, the future of terahertz (THz) communication systems, especially in hybrid setups, can be seen in the desire to advance these systems while eliminating their current limitations and making them fit for other applications. Presently, these limitations can be overcome by the upcoming technologies and strategies that will make THz communication more effective. The future of THz-FSO communication relies heavily on overcoming the inherent challenges posed by atmospheric absorption, dispersion, and security vulnerabilities. One of the most critical aspects is the accurate modelling of THz propagation characteristics in real-world atmospheric conditions. Recent studies have shown that, beyond 500 GHz, the atmospheric attenuation surpasses 100 dB/km, severely limiting long-range THz communications. However, Ma et al. (2024) [107] conducted an in-depth analysis of THz channel behavior under various atmospheric conditions, highlighting the significant impact of humidity, temperature variations, and oxygen absorption on the signal degradation and security performance of the system. Their findings emphasize that adaptive multi-band switching and hybrid THz-FSO-RF systems could mitigate propagation losses by dynamically selecting the optimal frequency bands based on environmental conditions.

### 7.1. AI-Driven Network Management

AI and ML techniques can significantly enhance the administration of hybrid THz-FSO networks. It may be concluded that climate-sensitive models derived from the collected data might allow for the real-time reorganization of the frequency range and reallocation of resources, thereby improving the network availability and rate. AI optimum control methods also reduce latency effects in time-critical applications like semi-/fully autonomous cars and telemedicine.

### 7.2. Energy-Efficient Systems

However, the big power consumption is a shortcoming of THz-FSO systems that is being constantly addressed from time to time. Improving signal processing algorithms to optimize the energy consumption and power-saving hardware components of these systems could greatly extend their life. Another study could embrace an energy harvester focusing on the extraction of energy from the ambient environment, like solar or oscillator energy, in creating self-powered hybrid wire-line/wireless communication nodes.

### 7.3. Advanced Materials for THz Devices

Further refinements of THz communication will depend on upcoming materials with better characteristics. For example, metamaterial and two-dimensional materials such as graphene are capable of enhancing the efficiency and tunability of THz transmitters and receivers. Such materials can also help develop compact as well as low-cost THz components, allowing this technology’s application in different areas.

### 7.4. Integration with Quantum Communication

The integration of THz-FSO communication systems with quantum communication technologies sheds light on a new approach to secure high-bandwidth networks. Putting QKD protocols into THz systems might offer improved security levels to applications that require high-level protection, like financial transactions and governmental uses.

### 7.5. Standardization and Regulation

To successfully deploy THz communication systems, the unified standardization of frequency bands and policies across nations is necessary. There is a need for various stakeholders, researchers, and policymakers to embark on joint initiatives to develop framework specifications that would enhance compatibility across various frontiers to allow optimal utilization of the spectrum.

### 7.6. Deep Space Communication

Improvements in THz-FSO systems may play a role in furthering future deep space communication architectures for high-data-rate and low-latency interplanetary platforms. This capability is inherent for further and subsequent space exploration and colonization missions, as colonization would require time-effective and secure communication.

## 8. Conclusions

This study standardizes the definitions of frequency bands, explicitly distinguishing between microwave (300 MHz–30 GHz), millimeter-wave (30 GHz–300 GHz), and terahertz (300 GHz–10 THz) communication systems to ensure consistency in performance comparisons and practical applications. THz communication systems are indeed the next generation beyond the current wireless communication systems, as they can offer unprecedented system data rates, high spectral efficiency, and promising use cases in fields including space exploration, beyond 5G or 6G communications, and secure communication. This paper presents a detailed study of THz communication technologies’ evolution, issues, and trends with reference to FSO and hybrid system implementation. The analysis of high-power THz sources shows that the development of new technologies such as quantum cascade lasers (QCLs), photonic mixers, and free-electron lasers (FELs) expands the possibilities of the THz generation even despite problems such as cooling needs and high costs of operation. THz-FSO utilization has been made more reliable through adaptive optics and advanced signal processing algorithms, showing the efficiency of using THz-FSO communication in dynamic conditions. These advances are supported by the application of machine learning to change beam orientations, construct signals, and employ methods of error dispersion to maintain proper communication when the environments are unfavorable. Integrating THz-FSO with optical and radio frequency communication systems has been proposed as a reasonable solution to the range and reliability issues of the existing THz systems. Using SDN and multi-band adaptive frameworks, these half-and-half designs allow standardized and systematic conveyance in the midst of outrageously good conditions like atmospheric babble. This review also highlights the special applications that are becoming possible for THz communication, especially in secure and military-related fields, due to its relatively small interception angle. Likewise, incorporating THz communication into 6G networks is expected to provide ultra-low latency high-capacity solutions in the next generation of applications, such as fully autonomous systems and holographic telepresence. Lastly, THz communication systems have the potential to profoundly change the practice of data transmission and provide new solutions to the continuous development of modem communication systems. However, to unlock its full potential, more interdisciplinary research activity that focuses on issues to do with energy conversion efficiency, materials, and integration into a system needs to be undertaken. With the cooperation of academia, industries, and regulators, THz technologies will open a new chapter of development in telecommunication, space communication, and secure networks, continuing the future communication paradigm into the 21st century.

## Figures and Tables

**Table 1:** Comparison of Communication Technologies.

Sr. No.	Microwave Communication [17,18]	Optical FSO Communication [1,7]	THz-FSO Communication [5,19]
1	Bandwidth is limited to the GHz range. Suitable for long-distance links but offers lower data rates.	Moderate bandwidth (100s of GHz) with higher data rates compared to microwave systems.	Extremely high bandwidth (THz range), enabling data rates beyond 1 Tbps.
2	Minimal atmospheric attenuation ensures stable links even under adverse conditions.	Moderate attenuation due to environmental factors like fog and rain.	High atmospheric attenuation caused by water vapor absorption.
3	Long transmission distances are supported by lower frequencies and minimal loss.	Medium-range communication is suitable for urban and controlled environments.	Short to medium-range communication requiring advanced alignment mechanisms.
4	Cost-effective due to well-established hardware technologies.	Higher hardware costs are driven by advanced optical components and alignment systems.	High costs due to nascent THz components like QCLs and high-power detectors.
5	Security is moderate due to wide beam propagation, making interception easier.	High security with narrow beamwidths reducing interception risks.	Very high security due to highly directional and narrow beams.

**Table 2:** Comparative analysis of high bandwidth across technologies.

Studies	Technology	Frequency Range	Bandwidth (GHz)	Applications
[23]	Microwave	300 MHz–30 GHz	1–10 GHz	IoT, mobile communication, satellite links
[24]	Millimeter-Wave	30 GHz–300 GHz	10–100 GHz	5G, short-range backhaul, automotive radar
[25]	Terahertz (THz)	300 GHz–10 THz	100 GHz–10 THz	6G, ultra-HD streaming, inter-satellite links
[26]	Technology	Frequency Range	Bandwidth (GHz)	Applications
[27]	Microwave	300 MHz–30 GHz	1–10 GHz	IoT, mobile communication, satellite links

**Table 3:** Comparative analysis of shorter wavelengths across technologies.

Studies	Technology	Frequency Range	Wavelength Range	Component Size
[31]	Microwave	300 MHz–30 GHz	1 mm–1 m	Bulky antennas
[32]	Millimeter-Wave	30 GHz–300 GHz	1 mm–10 mm	Compact antennas
[33]	Terahertz (THz)	300 GHz–10 THz	30 µm–1 mm	Ultra-compact devices
[21]	Technology	Frequency Range	Wavelength Range	Component Size
[34]	Microwave	300 MHz–30 GHz	1 mm–1 m	Bulky antennas

**Table 4:** Comparative analysis of higher data rates across technologies.

Studies	Technology	Data Rates (Gbps)	Applications	Key Challenges
[21]	Microwave Communication	10–100	IoT, mobile networks	Limited spectrum
[35]	Optical Communication	100–400	Datacenters, fiber-optic networks	High cost of deployment
[38]	THz Communication	1000–10,000	6G networks, inter-satellite links	Atmospheric attenuation
[39]	Photonic-Assisted Systems	1000+	High-speed backhauling	Limited scalability
[40]	Nanofabricated THz Systems	4000+	Real-time analytics, AR/VR	Complex integration

**Table 5:** Atmospheric absorption challenges in THz communication.

Studies	Challenge	Cause	Proposed Solution	Limitation
[16]	Signal attenuation	Water vapor absorption	Photonic crystal fibers	High fabrication cost
[54]	High absorption	Atmospheric composition	Low-loss materials	Limited bandwidth
[55]	Reduced range	Resonance of water vapor molecules	Frequency selection	Bandwidth constraints
[56]	Absorption peaks	Atmospheric pressure variations	Metamaterial-based designs	Manufacturing costs
[57]	Limited range	Scattering and absorption	Dynamic path optimization	Energy overhead
[58]	Variable losses	Environmental changes	Real-time sensing systems	Algorithm complexity
[57]	Bandwidth reduction	Selective frequency operation	Dynamic reconfigurations	System overhead
[59]	Environmental sensitivity	Temperature and pressure effects	Advanced atmospheric models	Limited precision in dynamic conditions

**Table 6:** Challenges in LOS alignment for THz-FSO communication.

Study	Challenge	Cause	Solution	Key Limitation
[48]	Misalignment	Turbulence, vibrations	Adaptive optics	High complexity
[49]	Precision requirement	Short wavelengths	Beam-steering mechanisms	Costly hardware
[50]	Environmental instability	Platform vibrations	Vibration compensation systems	Increased energy usage
[51]	Beam divergence	Large aperture size constraints	Phased array antennas	Complex manufacturing
[52]	Dynamic conditions	Moving platforms	Adaptive real-time controls	Hardware scalability
[53]	Atmospheric effects	Refraction and scattering	Dynamic path optimization	Computational overhead
[16]	Alignment drift	Structural vibrations	Feedback-based alignment	Delay in adjustments
[54]	High sensitivity	Shorter wavelengths	Precision beamforming	High computational requirements

**Table 7:** Hardware challenges in THz communication.

Parameter	Device Type	Value/Range	Challenge	Cause	Solution	Citation
Operating Temperature	Quantum Cascade Lasers (QCLs)	~77 K (Cryogenic Cooling)	Cooling requirements	High thermal dissipation in QCLs	Cryogen-free designs and better thermal management	[57]
	Difference-Frequency Generation QCLs (DFG-QCLs)	Room Temperature	Limited operational efficiency	Higher energy loss at room temp	Material advancements in active regions for better efficiency	[57]
Detector Sensitivity	Schottky Diode Mixers	~10–100 pW/Hz^0.5^	Limited responsivity	High noise levels in mixers	Noise-reduction techniques and improved materials	[59]
	Bolometers	Sub-pW Sensitivity	High cost	Expensive cryogenic cooling needs	Development of room-temperature bolometers	[60]
	Photoconductive Antennas	~100 fW/Hz^0.5^	Low efficiency	Carrier lifetime limitations	Advanced semiconductor material integration	[60]
Output Power	Quantum Cascade Lasers (QCLs)	~10 mW	Low power output	High threshold current	Novel gain media and cavity optimizations	[61]
	Photonic Mixers	~100 µW	Low conversion efficiency	Energy loss in photomixing process	Enhanced phase-matching techniques	[62]
	Free-Electron Lasers (FELs)	Watt-level Power Output	High power consumption	Large-scale accelerator requirements	Miniaturization and compact FEL designs	[62]
Modulation Bandwidth	Direct Modulation	Limited to GHz-range	Limited carrier transport	Device material constraints	High-speed heterostructures for improved modulation	[63]
	External Modulation	Extends to THz-range	Complexity in integration	Coupling losses in external modulators	Low-loss integration techniques	[63]

**Table 8:** Signal distortion challenges in THz communication.

Studies	Challenge	Cause	Solution	Limitation
[28]	High distortion	Atmospheric turbulence	Adaptive equalization	Computational cost
[12]	Scattering effects	Environmental factors	Beam-shaping techniques	Implementation complexity
[64]	High error rates	Signal distortion	Error-correction codes	Latency overhead
[65]	Beam divergence	Uneven wavefront distortion	Real-time sensing systems	Hardware costs
[66]	Environmental variability	Rapid atmospheric changes	Polarization techniques	Limited scalability
[67]	Turbulence impact	$\mathrm{High}\;C_{n}^{2}$ regions	Kolmogorov turbulence	Precision challenges
[68]	Range limitations	Attenuation and absorption	Enhanced receiver designs	Increased energy demand

**Table 9:** Advanced materials for THz-FSO communication.

Studies	Material	Applications	Advantages	Limitations
[43]	Graphene	THz modulators	High mobility, tunable properties	Fabrication scalability
[74]	2D materials	Photodetectors	Ultra-fast response	Integration challenges
[70]	Metamaterials	Beam shaping	High precision	High cost
[41]	Nanoparticles	Loss reduction in photonic fibers	Reduced dispersion	Limited bandwidth
[75]	Metamaterials	Polarization control	Enhanced stability	Complex design
[22]	ITO/SeO_2_	Optical modulators	Enhanced link performance	Modulation instability

**Table 10:** Integrated photonic devices for THz-FSO communication.

Studies	Device	Functionality	Advantages	Challenges
[23]	Quasi-optic modules	Wireless links	Ultra-broadband	Signal alignment
[28]	MZI-MRR circuits	Pulse processing	Programmability	Fabrication precision
[31]	Cascaded MIMO equalizers	Data integration	Enhanced bandwidth	Energy demand
[35]	Integrated antennas	Beamforming	Compact size	Thermal stability
[39]	Phase modulators	Signal modulation	High precision	Design complexity
[16]	Photonic waveguides	Light propagation	Low dispersion	Material losses

**Table 11:** Comparison of high-power THz sources.

Studies	Source	Advantages	Limitations	Applications
[12]	QCLs	Compact, high tunability	Cooling requirements	Long-range communication
[50]	Photonic Mixers	Broad bandwidth	Phase coherence issues	Spectroscopy, imaging
[52]	FELs	Extremely high power	Large size, high cost	Scientific research

**Table 12:** Comparison of adaptive optics technologies.

Studies	Technology	Advantages	Limitations	Applications
[91]	Wavefront correction	Improves Strehl ratio	Computational cost	Signal integrity
[92]	Real-time AO systems	Dynamic correction	Requires high-speed hardware	Long-distance communication
[93]	Phase-only SLMs	High-resolution phase control	Limited by modulation speed	Turbulence compensation

**Table 13:** Comparison of frequency bands in hybrid systems.

Study	Frequency Band	Advantages	Limitations	Applications
[104]	Microwave	Long-range, robust to weather	Limited data rates	Weather-independent communications
[105]	Millimeter-wave	Higher data rates, moderate range	Susceptible to rain attenuation	Short-range mobile networks
[106]	THz band	Ultra-high data rates, narrow beam	Short range, high attenuation	High-capacity backhaul
